# Contraceptive Use Before and After Gastric Bypass: a Questionnaire Study

**DOI:** 10.1007/s11695-015-1641-7

**Published:** 2015-03-06

**Authors:** Charlotte Ginstman, Jessica Frisk, Johan Ottosson, Jan Brynhildsen

**Affiliations:** 1 Department of Obstetrics and Gynaecology, University Hospital, Linköping University, 58185 Linköping, Sweden; 2 Department of Clinical and Experimental Medicine, Linköping University, Linköping, Sweden; 3 Department of Surgery and Department of Clinical and Experimental Medicine, Linköping University, Norrköping, Sweden; 4 Department of Surgery, Örebro University, Örebro, Sweden

**Keywords:** Gastric bypass, Pregnancy, Contraception, Obesity

## Abstract

**Background:**

At present, women are recommended to avoid pregnancy 12–18 months after bariatric surgery. Our aim in this study was to describe patterns of contraceptive use before and after gastric bypass in Sweden, and to describe the contraceptive counseling given preoperatively to women undergoing gastric bypass.

**Methods:**

In October 2012, a questionnaire was sent to 1000 Swedish women who all had undergone gastric bypass during 2010. The women had been included in the Scandinavian Obesity Surgery Register at time of surgery. The main outcome measures were patterns of use of contraception before and after bariatric surgery.

**Results:**

The response rate was 57 %. The most commonly used contraceptive methods were intrauterine devices, 29 % preoperatively and 26 % postoperatively even though there was a postoperative switch from the copper intrauterine device to the levonorgestrel intrauterine system. Thirty percent did not use any contraceptive during the first 12 months after surgery. Sixty percent of the responders were aware of the recommendations to avoid pregnancy after surgery.

**Conclusions:**

Many women who undergo bariatric surgery are not using any contraceptive method despite the recommendation that they should avoid pregnancy for at least 12 months. There is a great need to improve contraceptive counseling for this growing group of women.

## Introduction

Fifty-one percent of the adults in Sweden have been reported to be either overweight or obese. The proportion of obese Swedish women, i.e., with BMI >30 kg/m^2^ (BMI = body mass index) has increased from 11 to 14 % between 2004 and 2012 [1]. Bariatric surgery is considered to be the most effective treatment for morbid obesity [2], and the number of operations in Sweden has increased dramatically from nearly 1000 in 2004 to 7700 in 2013 [3]. The number of operations performed as a percentage of the total population is relatively high in Sweden, 0.09 %, in comparison with Japan 0.0001 % or USA/Canada 0.03 % [4]. Ninety-two percent of all bariatric surgery in Sweden is publically financed. Surgery is offered if no contraindications are present to those seeking help with BMI >35 kg/m^2^. The indication for bariatric surgery is even stronger if a concomitant disorder such as diabetes, hypertension, joint disease, and severe sleep apnea is present.

The leading surgical technique is gastric bypass (Roux-en-Y gastric bypass, RYGB), which constitutes 92 % of all bariatric surgery in Sweden [3]. Women constitute 76 % of all patients undergoing gastric bypass in Sweden, and the median age for patients is 41 years [3]. Women undergoing bariatric surgery in Sweden as well as in other Western countries including the USA are most often advised not to become pregnant for 12–24 months postoperatively to avoid potential risks of fetal complications such as an increased risk of premature birth and children born small for gestational age [5–7] and to avoid suboptimal weight loss [8, 9]. Children born by RYGB mothers have a higher incidence of lower birthweight (SGA, small for gestational age), but there does not seem to be an increased risk of malformations in pregnancies following surgery [10]. Kjaer et al., however, showed no differences in pregnancy outcomes between children born within or after the first postoperative year [11]. Theoretically, the malabsorptive intestinal surgical procedures used may affect the absorption of medicine taken orally [12]. If, and how, the absorption of oral contraceptives is affected by the gastric bypass method has not been studied. Victor et al. in 1987 found significantly lower plasma levels of both norethisterone and levonorgestrel postoperatively, suggesting a reduced absorptive capacity for progestogens 1 to 3 years after jejunoileal bypass [13]. However, this procedure is much more extensive than gastric bypass. Retrospective studies [14, 15] have indicated a small increase in risk of contraceptive failure in obese women, but this could not be confirmed in a prospective study [16]. A small study from the USA showed that 16 % of the women who underwent bariatric surgery did not use effective contraceptive methods despite their knowledge of the importance of avoiding pregnancy during 12–18 months postoperatively [17]. To our knowledge, this is the only published study concerning this matter. We aimed to in a larger sample describe how women having bariatric surgery perceived the contraceptive counseling they were given before the procedure and to determine patterns of contraceptive use before and after the surgery in these women.

## Methods

The Scandinavian Obesity Surgery Register (SOReg) provided names and personal identification numbers of 1000 women aged 18–45 years, who had undergone bariatric surgery in Sweden during 2010. Correct addresses could be retrieved to 987 of these women, and a postal questionnaire was sent to these women. The questionnaire comprised questions regarding educational level, smoking habits, pre- and postoperative weight, and history of childbirth and pregnancies both before and after the operation. Other questions concerned previous and present contraceptive use, contraceptive counseling, and recommendations concerning avoidance of pregnancy after bariatric surgery. We also asked questions regarding type and frequency of stool as frequent, loose stool might affect uptake of pharmacological agents. Moreover, questions on oophorectomy and hysterectomy were included. Data on comorbidity were retrieved from SOReg.

### Validation Procedure

The questionnaire was validated with a “think-aloud” cognitive interviewing technique [18]. Six women who all recently had undergone gastric bypass answered the questionnaire orally, face to face, together with one of the researchers (CG), and all comments on the questionnaire was noted. This procedure identified some questions that needed remodeling. After modification of the questionnaire, the validity was considered as good.

For comparison, SOReg provided preoperative data on smoking habits, weight, and BMI.

The questionnaires were coded, which enabled us to send a reminder to those women who had not answered after 4 weeks, and a second reminder was sent after another 4 weeks. When the codes had been eliminated, the questionnaires were optically scanned into the computer. Optical scanning was checked manually, and when the first 10 questionnaires had shown total agreement between optical and manual reading, the procedure was accepted.

Since all women did not answer every question, some of the results have different numbers of participants. Proportions may sum >100 % in cases where participants were permitted to choose more than one option.

### Statistics

SPSS version 21.0 was used to analyze data, mostly in forms of descriptive statistics. Chi-square test was used for comparisons between groups.

## Results

In total, 563 women answered the questionnaire, and consequently, the response rate was 57 %. Two women returned a blank questionnaire. Sixty-four percent had the operation more than 2 but less than 3 years earlier. The remaining 36 % had the surgery between 1 and 2 years earlier. Nineteen percent had never been pregnant, and 28 % were nulliparous. Median weight before and after surgery was 122 and 75 kg, respectively (Table 1).

**Table 1:** Characteristics of the 563 women who answered the questionnaire. The number may vary because all women did not answer all questions. Values are presented as median (range) or numbers (%)

Characteristics		
Age (years)	36	(22–43)
Preoperative weight (kg)	122	(80–200)
Present weight (kg)	75	(47–164)
Preoperative BMI (kg/m^2^)	43.6	(29.7–73.5)
Present BMI (kg/m^2^)	27.1	(18.4–64.1)
Parity	2	(0–6)
Current partner		
None	100	(18 %)
No cohabitating partner	38	(6.8 %)
Cohabitating partner	419	(75.2 %)
Highest diploma		
High school	45	(8.0 %)
College	365	(65.3 %)
Graduate school	149	(27.7 %)
Professional situation		
Working	389	(72 %)
Unemployed	37	(6.9 %)
Student	33	(6.1 %)
Others (housewife, sick leave, parental leave)	81	(15.0 %)

*BMI* body mass index

There were no differences in weight loss during the first postoperative year in relation to the contraceptive method the women used during this time (data not shown).

Sixty-seven percent of the women used some kind of contraceptive method preoperatively (Table 2). Eighty percent of these women were satisfied with their preoperative contraceptive method.

**Table 2:** Contraceptive methods used before and after surgery

Contraceptive^a^	12 months before, *n* = 563	1–2 years after, *n* = 563	Sign.^c^	1–2 years after, *n* = 563
	*n* (%)	*n* (%)		*n* (%)
Longer-acting reversible contraceptives				
Copper intrauterine device	77 (13.7)	61 (10.9)	NS	40 (7.1)
Lng IUS^b^	86 (15.3)	104 (18.5)	NS	108 (19.2)
Implant	21 (3.7)	23 (4.1)	NS	14 (2.5)
Short-acting hormonal contraceptives				
Any oral contraceptive	130 (23.1)	87 (15.5)	*p* = 0.001	53 (9.4)
Combined oral contraceptives	43 (7.6)	29 (5.2)	NS	22 (3.9)
Desogestrel progestin-only pill	59 (10.5)	46 (8.2)	NS	28 (5.0)
Other progestin only pills	28 (5.0)	12 (2.1)	*p* = 0.01	3 (0.5)
Vaginal ring	6 (1.1)	13 (2.3)	NS	7 (1.2)
Patch	0 (0)	5 (0.9)		3 (0.5)
Other contraceptives				
Depo-Provera®	34 (6.0)	32 (5.7)	NS	29 (5.2)
Condoms/other	110 (19.5)	109 (19.4)	NS	78 (13.7)
None	182 (32.3)	168 (29.9)	NS	210 (37.2)

^a^Participants could choose more than one option if they had changed method during the given period of time

^b^Levonorgestrel intrauterine system

^c^12 months prior to surgery vs. 12 months postsurgery. Chi-square test

Progestin-only pills were the most commonly used oral contraceptive during the preoperative period. The use of various oral contraceptives significantly declined after surgery, but approximately 10 % were still using an oral contraceptive at the time of answering the questionnaire, i.e., more than 1 year after surgery (Table 2). Intrauterine contraception was the most commonly used method both before and after surgery. A trend to a shift from use of copper intrauterine devices in favor of levonorgestrel intrauterine systems was noted, however not significant (Table 2).

Almost every fourth woman (24.8 %) actively stated that she did not receive any advice that she should avoid pregnancy postoperatively, and 14.8 % did not remember whether or not they had received such advice or not. A majority (96.8 %) of the women who received information had been told they should avoid pregnancy for 12–24 months after surgery. The main sources of this information were midwives or gynecologists. The women stated that they had to arrange an appointment themselves. Eighteen percent stated they could have benefited from more contraceptive counseling at the time of surgery.

Almost one third of the women had not used any contraception at all during the first year following surgery. Among the women who presently used contraception, a majority (82 %) were satisfied with their contraceptive method (Table 2).

Twenty-five percent had become pregnant after surgery, and 12 % were actively trying to become pregnant when they answered the questionnaire. Almost 3 % reported becoming pregnant postoperatively in spite of using contraception properly, but from the information available, it was not possible to determine what methods these women had used at the time of conception or how far after the surgery it had happened. A vast majority of the women (98 %) had normal frequencies of stool ranging from three times per day up to only every other day.

## Discussion

This study reflects the experience of a large sample of women of fertile age who had undergone bariatric surgery. A substantial number of these women stated that they had received insufficient advice about avoiding pregnancy. A majority also stated that they received no or insufficient contraceptive counseling.

The Swedish guidelines recommend avoiding the combination of obesity and use of combined hormonal contraceptives [19], as this has been shown to increase the risk of venous thromboembolism [20–22]. Also, the American College of Obstetricians and Gynecologists (ACOG) state that due to the increased thromboembolic risk in obese women “consideration should be given to progestin-only and intrauterine methods when counseling obese women regarding contraceptive choices” [23]. It is therefore noteworthy and alarming that almost every tenth woman had used combined hormonal contraceptives at some time within 1 year prior to surgery. The percentage of women using combined hormonal methods was even higher, 27 %, in the USA in the study by Mody et al. [17].

Sixty-seven percent of the women used any contraceptive method which is slightly lower than in the general Swedish population (72 %, Helena Kopp-Kallner et al., Karolinska Institutet, Stockholm, unpublished data). The use of oral contraceptives remains relatively high after gastric bypass, which is surprising, since present knowledge of how surgery affects the uptake and effect of oral contraceptives after surgery is limited, but previous data indicate a decrease in the absorption of oral contraceptives. Further pharmacokinetic studies are needed in this field to provide a basis for improving contraceptive counseling for this group.

Smoking was reported equally frequent postoperatively (16 %) as compared with preoperative data from SOReg (13.5 %), which indicates that the preoperative smoke restriction is only temporarily followed.

A substantial number of the women did not receive any contraceptive counseling. These findings are similar to those of Mody et al. [17] and emphasize the need for improved contraceptive counseling for women undergoing gastric bypass surgery, both in a European and American perspective.

Since obese women often suffer from obesity-related infertility, it was not surprising that the overall usage of contraception declined 1 year after surgery. This pregnancy-wish may also partly explain why 30 % did not use contraception at all during the 12-month period postoperatively, a percentage almost double with what was reported earlier by Mody et al. [17]. Failure to use contraception may also be explained by the fact that as many as 25 % cannot recall receiving this recommendation.

The number of women becoming pregnant after gastric bypass in spite of contraceptive use does not seem to be higher than expected in any group of women during the 1 to 2 years of follow-up. We found no difference in weight loss after surgery between women using different kinds of contraceptives. These observations must be viewed with caution as the groups differed in size range between 5 and more than 100.

This study reflects the contraceptive use and presents information on the insufficient contraceptive counseling in relation to bariatric surgery in a large sample of obese women using a validated questionnaire. To our knowledge, this is the largest study concerning contraceptive counseling for women who undergo bariatric surgery. Some of the information from the questionnaire could be compared with the prospectively gathered data in SOReg, and the self-reported preoperative BMI with a median of 43.6 (Table 1) correlated well with data of preoperative BMI from SOReg (median 42.3).

The low response rate, however, suggests that the interpretations must be viewed with caution. Dropout analysis showed that the responding group was similar to the non-responding group of women in terms of preoperative BMI, comorbidity, and age. According to data from SOReg, there were slightly more smokers in the non-responding group, but we do not believe this has biased our results.

Still many authorities recommend a woman undergoing bariatric surgery to avoid pregnancy the first year (s) after the operation. However, a recent Danish study [24] was not able to demonstrate a relation between the “surgery-to-conception” interval and the fetal outcome, and it might be speculated that the current recommendations might be revised. Rapid weight loss have been shown to increase the serum levels of persistent organic pollutants, normally stored in body fat [25], which theoretically could be a strong reason to postpone pregnancy until the weight is stabilized. But irrespective if a revision will be necessary or not, both the present study and the previous study by Mody et al. [15] show a need of improved contraceptive counseling for obese women undergoing bariatric surgery.

## Conclusion

We identified a great need to provide improved contraceptive counseling for this growing group of women undergoing gastric bypass surgery. Health-care professionals involved in bariatric surgery need to emphasize the recommendations concerning contraception and, if needed, refer the patients to a gynecologist preoperatively for contraceptive counseling. We suggest that standard written information on advice concerning postoperative pregnancies/contraception should be handed out by the operating center to every individual considering undergoing bariatric surgery.
